# Kallikrein-Related Peptidase 5 Is Associated with Tumor Progression, KRT19 Expression, and Distinct Molecular Programs in High-Grade Serous Ovarian Cancer

**DOI:** 10.3390/cancers18203387

**Published:** 2026-10-20

**Authors:** Sophia Bielesch, Antonia Blechschmidt, Georgia Lachanioti, Johanna Moeller, Gregor Bielesch, Sophia Fella, Dalila Darmoul, Stefan Tholen, Oliver Schilling, Holger Bronger, Marion Kiechle, Viktor Magdolen, Tobias Dreyer

**Affiliations:** 1 Department of Gynecology and Obstetrics, TUM University Hospital, 81675 Munich, Germany; sophia.bielesch@tum.de (S.B.); antonia.blechschmidt@tum.de (A.B.); georgialachanioti@outlook.com (G.L.); johanna.moeller@tum.de (J.M.); sophia.fella@tum.de (S.F.); holger.bronger@tum.de (H.B.); marion.kiechle@tum.de (M.K.); viktor.magdolen@tum.de (V.M.); 2 Laboratory of Clinical Biochemistry—Molecular Diagnostics, Second Department of Pediatrics, “P. & A. Kyriakou” Children’s Hospital, School of Medicine, National and Kapodistrian University of Athens, 11527 Athens, Greece; 3 Department of Trauma Surgery, TUM University Hospital, 81675 Munich, Germany; gregor.bielesch@mri.tum.de; 4 Institut de Biologie Paris Seine, CNRS UMR8263-INSERM U1345, Sorbonne Université, 75005 Paris, France; dalila.darmoul@inserm.fr; 5 Institute for Surgical Pathology, Medical Center, University of Freiburg, 79098 Freiburg, Germany; stefan.tholen@uniklinik-freiburg.de (S.T.); oliver.schilling@uniklinik-freiburg.de (O.S.); 6 German Cancer Consortium (DKTK), Partner Site Munich (A Partnership Between DKFZ and Technical University of Munich), 69120 Heidelberg, Germany

**Keywords:** kallikrein-related peptidase 5, cytokeratin 19-regulation, ovarian cancer, spheroid formation, cholesterol homeostasis, CXCL9

## Abstract

**Background/Objectives**: High-grade serous ovarian cancer (HGSOC) is characterized by peritoneal dissemination and ascites formation. Kallikrein-related peptidase 5 (KLK5) has been linked to aggressive disease. Building on evidence linking KLK4–7 to keratin 19 (KRT19), we hypothesized that a KLK5–KRT19 axis contributes to tumor progression and investigated this potential axis alongside broader KLK5-associated tumor-cell, metabolic, signaling, and microenvironmental mechanisms. **Methods**: KLK5-overexpressing ovarian cancer models were characterized using functional assays (adhesion, migration, spheroid formation), KRT19 expression analysis, proteomics, and gene set enrichment analysis. The putative KRT19–KLK5 axis was further investigated in vivo, in silico, and by immunohistochemistry. KLK5-dependent tumor progression was assessed in vivo, including survival and ascites formation, with quantification of CXCL9 in ascitic fluid. Clinical relevance was evaluated by immunohistochemistry in an HGSOC cohort. **Results**: In vitro, KLK5 overexpression induced KRT19 expression and enhanced spheroid formation, whereas effects on adhesion and migration were limited. Proteomic analysis identified KLK5-associated alterations in cholesterol homeostasis, coagulation, and mTORC1 signaling. In the in vivo mouse model, KLK5 overexpression was associated with reduced survival and increased ascites formation, and KLK5 induced intratumoral KRT19 expression. KLK5 and KRT19 protein levels were positively associated in patient tumors and primary ascites-derived tumor cells. In ascites, KLK5 overexpression was associated with reduced CD3+ cell abundance and lower CXCL9 concentrations. **Conclusions**: KLK5 promotes HGSOC progression. Its association with KRT19 expression, altered cholesterol homeostasis and mTORC1 signaling, reduced CD3+ cells and CXCL9 in ascites suggests that KLK5 contributes to both tumor-cell and immune features of HGSOC.

## Simple Summary

High-grade serous ovarian cancer is the most common and aggressive form of ovarian cancer and frequently spreads throughout the abdominal cavity. A better understanding of the biological factors progressing the disease is needed to identify new biomarkers and potential therapeutic targets. In this study, we investigated the role of the kallikrein-related peptidase 5 (KLK5), which is frequently elevated in ovarian cancer. We found that high KLK5 levels were associated with increased expression of keratin 19, a protein linked to tumor progression, and with enhanced formation of tumor cell clusters. KLK5 was also associated with changes in cholesterol-related cellular processes and with features suggesting reduced anti-tumor immune activity. Moreover, high KLK5 levels were linked to poorer survival in patients and a syngeneic mouse model. These findings provide a broader understanding of KLK5 in ovarian cancer and support further investigation of KLK5 as a potential biomarker and therapeutic target.

## 1. Introduction

Ovarian cancer represents the eighth leading cause of cancer-related mortality among women worldwide. Approximately 90% of ovarian cancers are epithelial in origin, with high-grade serous ovarian cancer (HGSOC) constituting the most prevalent and lethal histological subtype, accounting for 70–80% of epithelial ovarian malignancies [1]. Despite significant advances in surgical techniques and chemotherapeutic regimens, HGSOC remains one of the most aggressive gynecological malignancies. Nearly 80% of patients are diagnosed at advanced disease stages (FIGO III–IV), and five-year overall survival rates remain poor, ranging from only 10–40%. The aggressive clinical course of HGSOC, characterized by rapid peritoneal dissemination, early development of chemoresistance, and high recurrence rates, underscores the urgent need to identify novel molecular drivers of disease progression [1,2,3].

**Figure 1:**
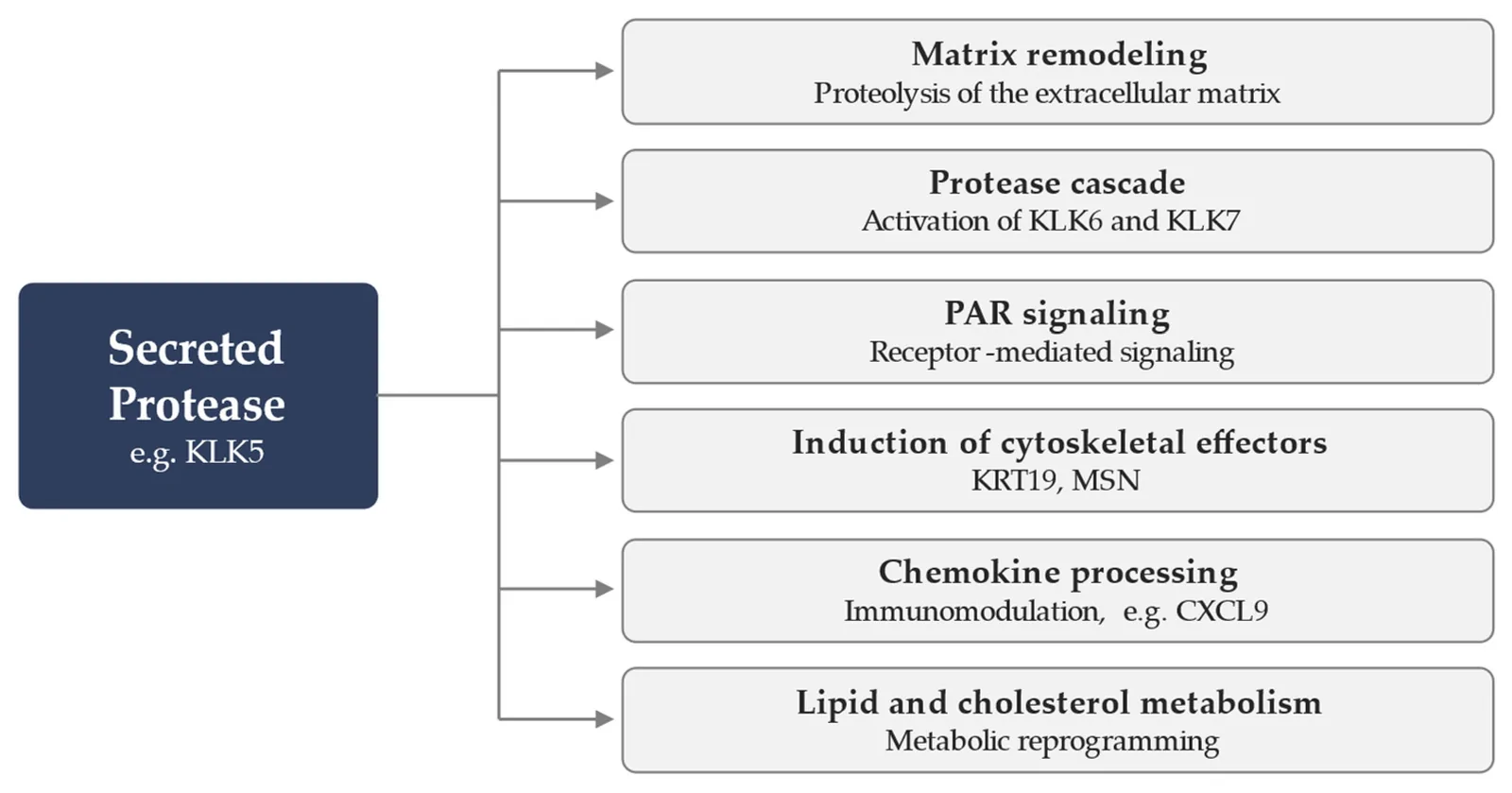
Proposed mechanisms by which KLKs influence tumor biology.

Kallikrein-related peptidases (KLKs) comprise a family of 15 secreted serine proteases encoded by the largest contiguous protease gene cluster in the human genome, located on chromosome 19q13.3–4 [4,5]. Through their proteolytic activity, KLKs regulate a broad range of physiological processes, including tissue remodeling, renal function, neural plasticity, and skin desquamation [5,6,7]. In cancer, KLKs have emerged as key modulators of the tumor microenvironment, influencing multiple hallmarks of malignancy via both proteolytic and non-proteolytic mechanisms [8,9]. Functionally, KLKs are proposed to influence tumor progression through several mechanistically distinct, and not necessarily interdependent, routes. The most relevant mechanisms suggest proteolytic remodeling of the extracellular matrix, activation of downstream proteases, PAR-mediated receptor signaling, induction of cytoskeletal effectors, including KRT19, processing of immunomodulatory chemokines, and reprogramming of lipid and cholesterol metabolism (Figure 1) [9,10,11,12,13,14,15,16,17,18].

Several KLKs, most prominently KLK4, KLK5, KLK6, and KLK7, are markedly overexpressed in HGSOC and consistently associated with adverse clinical outcomes [13,16,19,20,21,22]. It was shown that combined overexpression of KLK4–7 in ovarian cancer cells significantly increased tumor burden in vivo, enhanced invasive capacity, and conferred resistance to paclitaxel chemotherapy [13,19]. Notably, these studies identified keratin 19 (KRT19) as a major downstream target of KLK4–7, implicating a regulatory axis linking KLK proteases to cytoskeletal remodeling and cellular plasticity in HGSOC [13]. While the combined effect of KLK4–7 on KRT19 expression and tumor-relevant pathways has been established, the individual contributions of each KLK to tumor progression and KRT19 regulation remain to be defined. In this context, KLK5 emerged as a compelling candidate due to its position in the proteolytic cascade as a potential activator of KLK6 and KLK7 and thereby potential amplifier of the KLK-dependent proteolytic signaling [23,24]. KLK5 is a trypsin-like protease best known for its physiological role in epidermal homeostasis, where it mediates skin desquamation [25,26]. Under normal conditions, KLK5 activity is tightly regulated by endogenous inhibitors such as lymphoepithelial Kazal-type-related inhibitor [27], and disruption of this balance results in inflammatory skin disorders, including Netherton syndrome and atopic dermatitis [25,26,28]. In addition to its physiological and pathophysiological roles in the skin, increasing evidence suggests that KLK5 is also involved in tumor development and progression through several mechanisms mentioned above (Figure 1) [16]. How KLK5 may influence those tumor-relevant programs and what functional and clinical consequences arise have not yet been systematically addressed. We therefore set out to delineate the KLK5-dependent tumor biology of HGSOC, spanning tumor cell behavior, the cellular proteome, the ascitic immune milieu, and in vivo disease progression.

In the following introductory sections, we will introduce the tumor-promoting pathways that emerged from our analyses as particularly relevant to this research. KRT19, the downstream target identified in this work, is a type I intermediate filament protein that forms obligate heteropolymers with type II keratins, thereby providing structural integrity to epithelial cells [29]. Our group previously showed that KRT19 induction enhanced epithelial traits in mesenchymal HGSOC tumor cell populations, both in vitro and ex vivo, thereby promoting increased cell–matrix adhesion, enhanced migratory capacity, and efficient spheroid formation, ultimately facilitating resistance to anoikis [30]. Across multiple cancer types, including ovarian cancer, KRT19-positive tumors are consistently associated with increased invasiveness, metastatic potential, and poorer clinical outcomes [31,32,33,34]. Given the critical role of KRT19 in HGSOC and other cancers, elucidating the mechanisms underlying its increased expression may open avenues for developing future therapeutic strategies. While KRT19-driven plasticity promotes tumor cell dissemination and survival in suspension, successful peritoneal spread also requires evasion of the local immune surveillance operating within the ascitic compartment.

The peritoneal microenvironment of HGSOC is shaped by a distinct chemokine milieu that influences anti-tumor immunity. Among these, the interferon-γ–inducible CXCR3 ligand CXCL9 mediates the chemotactic recruitment of tumor-infiltrating lymphocytes, and its high expression is associated with increased T-cell infiltration and approximately doubled overall survival in HGSOC patient cohorts [35]. In malignant ascites, macrophage-derived CXCL9 correlates with the accumulation of effector-memory CD8+ T cells and prolonged relapse-free survival, underscoring the compartment-specific prognostic weight of this chemokine [36]. Consistent with a causal, tumor-suppressive role, forced CXCL9 expression in syngeneic murine models drives T-cell accumulation, delays ascites formation, prolongs survival, and can sensitize otherwise resistant tumors to anti–PD-L1 therapy [37]. KLKs are increasingly recognized as regulators of tumor immune suppression, including through direct proteolytic processing of chemokines [9,38,39]. Given that KLK5 belongs to the KLK4–7 cluster, which is frequently overexpressed in ovarian cancer and known to reshape the secreted proteome of ovarian cancer cells, it is biologically plausible that KLK5 modulates the CXCL9 axis, thereby contributing to the immunosuppressive ascitic microenvironment characteristic of HGSOC [40]. Whether KLK5 in fact modulates this axis in HGSOC, however, remains unknown. Reprogramming of the immune microenvironment does not occur in isolation but is intimately linked to tumor cell metabolism, which fuels the signaling and secretory programs underlying microenvironmental remodeling.

More recently, KLKs have been linked to cellular metabolic state, including pathways that govern lipid and cholesterol homeostasis. KLK5 physiologically operates within the lipid-rich matrix of the stratum corneum, where it initiates desquamation, and in breast cancer cells, its re-expression has been shown to modulate the mevalonate pathway, affecting cellular cholesterol content, fatty acid synthesis, and isoprenoid-dependent protein prenylation [15,26,41]. Although cholesterol and lipid metabolism are increasingly recognized as regulators of tumor cell behavior through their crosstalk with signaling axes such as PI3K/AKT/mTORC1, the interplay between KLK5 and lipid metabolic pathways in HGSOC remains to be characterized [42,43].

Taken together, these lines of evidence suggest that HGSOC progression is shaped by the interplay of various mechanisms, including KRT19-mediated cytoskeletal plasticity, an immunosuppressive ascitic chemokine milieu, and altered lipid and cholesterol metabolism, rather than by any single mechanism in isolation. This convergence raises the possibility that an upstream protease might regulate a well-defined downstream target, such as KRT19, and further contribute to the broader remodeling of the immune and metabolic tumor microenvironment, a role for which KLK5 has emerged as a particularly compelling candidate.

## 2. Materials and Methods

### 2.1. Immunohistochemical Staining

Five-micrometer sections were cut from formalin-fixed, paraffin-embedded tissue microarrays, mounted on glass slides, deparaffinized using xylene and graded ethanol, and subsequently rehydrated. For KLK5 staining, heat-induced epitope retrieval was performed in citrate buffer (pH 6.0) using a pressure cooker for 4 min after the second pressure indicator ring had been reached. Endogenous peroxidase activity was blocked with 3% hydrogen peroxide in distilled water for 20 min at room temperature. Sections were subsequently incubated with porcine serum (Dako; catalog no. X0901; 1:100 dilution; Glostrup, Denmark) for 20 min at room temperature to block nonspecific binding. Sections were incubated overnight at 4 °C with a goat polyclonal anti-KLK5 antibody (R&D Systems; catalog no. AF1108; lot no. XDT016031; 1:200 dilution; Minneapolis, MN, USA) diluted in Antibody Diluent (Zytomed; catalog no. ZUC025-500; Berlin, Germany). This was followed by incubation with a mouse anti-goat secondary antibody (Jackson ImmunoResearch; catalog no. 205-005-108; lot no. 122266; 1:200 dilution; West Grove, PA, USA) for 30 min at room temperature. Detection was performed using an HRP polymer detection system (One Step Polymer, Zytomed; catalog no. ZUC053-100; Berlin, Germany) for 30 min at room temperature. Staining was visualized using DAB High Contrast (Zytomed; catalog no. DABplus-500; Berlin, Germany) for 8 min at room temperature, followed by hematoxylin counterstaining. For KRT19 and CD3 staining, sections were incubated for 1 h at room temperature with an anti-KRT19 antibody (Abnova; catalog no. MAB10725; clone[GB2.1]; 1:200 dilution; Taipei, Taiwan) or an anti-CD3 antibody (DCS Diagnostics; clone SP7; catalog no. CI597C01; 1:100 dilution; Hamburg, Germany), respectively. Primary antibody incubation was followed by HRP polymer detection (Zytomed; Berlin, Germany) for 30 min and DAB visualization (Zytomed; Berlin, Germany) for 8 min, followed by hematoxylin counterstaining. Tris-buffered saline containing Tween-20 (TBS-T) was used for washing between steps. Immunohistochemical staining was evaluated using a semi-quantitative digital scoring approach as previously described [44], using ImageJ with the IHC Profiler Plugin (version 1.54p). Evaluation was performed by two independent observers. The slides were evaluated in a blinded manner, and only tumor regions were analyzed. A consensus of tumor regions was achieved between the two observers. For the analysis, the mean pixel intensity was used, and the mean of both observer values was used for further analysis.

### 2.2. Cell Culture

The HGSOC cell lines OV-MZ-6 (RRID: CVCL_4005, obtained from Möbus et al. [18]) and Kuramochi (RRID: CVCL_1345, obtained from JCRB Cell Bank JCRB0098) were cultured at 37 °C in a humidified atmosphere containing 5% CO_2_. OV-MZ-6 cells were maintained in DMEM (Gibco) supplemented with asparagine (0.004%)/arginine (0.01%). Kuramochi cells were cultured in RPMI-1640 (Gibco) supplemented with 10% FCS. Cells were grown in T-75 flasks and passaged at 80% confluence using 0.1% EDTA in PBS. Cell line authentication was performed by short tandem repeat profiling (Eurofins), and regular mycoplasma testing was conducted using Mycostrips^®^ (InvivoGen, San Diego, CA, USA).

### 2.3. Stably Transfected Cell Lines

OV-MZ-6 and Kuramochi cells were seeded in 6-well plates and transfected at approximately 70% confluence using Lipofectamine 3000 (Thermo Fisher Scientific, Waltham, MA, USA) with 2 or 4 µg of pRcRSV-KLK5 plasmid or the corresponding pRcRSV empty vector. After 6 h, the FCS-free transfection medium was replaced with complete medium containing FCS. Upon reaching confluence, cells were transferred to 10 cm culture dishes and selected with 0.5 g/L G418 (geneticin). Individual colonies were isolated, expanded, and stable transgene expression was confirmed by reverse transcription quantitative PCR (RT-qPCR). For syngeneic experiments, the mouse OC cell line (ID8 Trp53-/-) was transduced with lentiviral particles encoding either pSMPUW-Klk5 or the corresponding pSMPUW-empty vector, and cells were selected with G418.

### 2.4. Syngeneic Mouse Experiment

Animal experiments were approved by the Government of Upper Bavaria (ROB) under license Vet.: 2-22-135 and conducted in accordance with animal protection guidelines (TUM University Hospital). A total of 8 × 10^6^ ID8 Trp53-/- tumor cells suspended in DPBS were intraperitoneally injected into female eight-week-old C57BL/6JRj mice purchased from Charles River Laboratories, Wilmington, MA, USA. Mice were housed under specific pathogen-free conditions in individually ventilated cages with no detectable specific pathogens. Animal housing and handling were performed in accordance with the German Animal Welfare Act (Tierschutzgesetz) and the Federation of European Laboratory Animal Science Associations (GV-SOLAS) regulations. Mice were sacrificed upon signs of ascites and clinical symptoms (poor general condition, abnormal social behavior, or signs of anemia). Euthanasia was performed using isoflurane anesthesia followed by cervical dislocation. After paracentesis, the peritoneal cavity was opened, and all visible tumor nodules, mesentery, and ovarian tissue were excised. Animals were randomized before tumor inoculation.

### 2.5. Patient Samples

Tumor tissue specimens from 223 patients with accessible survival data with advanced-stage HGSOC were collected between 1990 and 2014 from the biobank of the Department of Obstetrics and Gynecology and the Institute of Pathology at the TUM University Hospital (Technical University of Munich). In 188 cases, follow-up data about recurrent events were available and included. Median follow up time was 43 months. Available events included for KLK5low/KLK5 high (OS: 126/36; PFS: 135/27). The study was approved by the local Ethics Committee (Faculty of Medicine, Technical University of Munich; project no. 491/17 S), and written informed consent was obtained from all patients. All patients underwent standard stage-appropriate primary radical debulking surgery followed by platinum-based adjuvant chemotherapy in combination with paclitaxel. No patient received neoadjuvant therapy. Clinical parameters, including age, ascites volume, residual tumor burden, and FIGO stage, were recorded (Table S1).

### 2.6. RNA Extraction and RT-qPCR

Total RNA was isolated using the NucleoSpin RNA Plus Kit (Macherey-Nagel, Düren, Germany). RNA concentration and purity were assessed using a NanoPhotometer^®^ N60 (Implen GmbH, Munich, Germany). Complementary DNA (cDNA) synthesis was performed using the RevertAid cDNA Synthesis Kit (Thermo Fisher Scientific). RT-qPCR was conducted using TaqMan assays (Thermo Fisher Scientific: human: *HPRT* (Hs02800695_m1), *KRT19* (Hs00761767_m1), *KLK4* (Hs00191772_m1), *KLK5* (Hs01548153_m1), *KLK6* (Hs00160519_m1), *KLK7* (Hs00192503_m1), KRT19 (Hs00761767_m1); mouse: *Hprt* (Mm03024075_m1), *Klk5* (Mm01203811_m1), *Krt19* (Mm00492980_m1)) and Blue Probe RT-qPCR Kit (Biozym, Hamburg, Germany). Cycling conditions were as follows: 95 °C for 2 min, followed by 40 cycles of 95 °C for 5 s and 60 °C for 25 s. Ct values were normalized to hypoxanthine-guanine phosphoribosyltransferase (HPRT) as an internal reference gene to account for differences in cDNA input and amplification efficiency.

### 2.7. Dot Blot

Culture media were conditioned for 48 h by OV-MZ-6 cells stably overexpressing KLK5 or carrying an empty-vector control. Supernatants (200 µL per sample) were applied to a nitrocellulose membrane using a mini dot blot chamber (Schleicher & Schuell, Dassel, Germany). The membrane was blocked for 1 h at room temperature with 5% milk in Tris-buffered saline containing Tween-20 (TBS-T), followed by overnight incubation with a mouse monoclonal anti-human KLK5 antibody (R&D Systems; catalog no. MAB1108; clone 193318; 1:100; Minneapolis, MN, USA) diluted in 2.5% milk in TBS-T. Subsequently, the membrane was incubated for 1 h at room temperature with an HRP-conjugated goat anti-mouse IgG (H + L) secondary antibody (Jackson ImmunoResearch Laboratories; catalog no. 115-035-003; 1:5000; West Grove, PA, USA) diluted in 2.5% milk in TBS-T. Signals were detected using enhanced chemiluminescence reagents (Millipore, Burlington, MA, USA).

### 2.8. Proliferation Assay

OV-MZ-6 cells stably overexpressing KLK5 and empty-vector controls were seeded at 50,000 cells per well. Cell growth was monitored using the PHIO Cellwatcher system through automated time-lapse imaging. Images were acquired over 150 h at 30 min intervals, and growth was quantified using Phio software (PHIOme, version 1.4.2). Analyses included 3 independent biological replicates per condition.

### 2.9. Adhesion Assay

First, 96-well plates were coated with fibronectin (2 µg/mL, Corning, New York, NY, USA), vitronectin (2 µg/mL), or collagen IV (1 µg/mL, Sigma-Aldrich, St. Louis, MO, USA) for 1 h at 37 °C, and thereafter, 2 × 10^4^ cells per well were seeded in adhesion buffer (0.5% BSA, 20 mM HEPES in cell culture media). Plates were incubated for 90 min with OV-MZ-6 cells and for 60 min with Kuramochi cells before adding the β-hexosaminidase substrate (Sigma-Aldrich). Afterward, the substrate reaction was stopped with EDTA/NaOH buffer. Absorbance was measured at 450 nm (Multiskan FC, Thermo Scientific, Vantaa, Finland). All data were normalized to empty vector control in the uncoated condition.

### 2.10. Trans-Well Migration Assay

Cell migration was assessed using a trans-well migration assay with 5 µm-pore polycarbonate membrane inserts (Corning). A total of 5 × 10^4^ cells were resuspended in 500 µL of serum-free medium and seeded into the upper chamber of the trans-well insert. The lower chamber was filled with 0.5 mL of medium supplemented with either 20% FCS or 0.4% BSA in PBS.

After incubation at 37 °C with 5% CO_2_ for 4 h in case of OV-MZ-6 cells and 22 h in case of Kuramochi cells, non-migrated cells were removed from the upper surface of the membrane. Migrated cells on the lower membrane surface were stained with 1 µg/mL 4′,6-diamidino-2-phenylindole (Invitrogen, Carlsbad, CA, USA) for 15 min. Cell migration was quantified by imaging four fields per membrane under a light microscope with QuPath. Representative pictures of stained nuclei can be found in the Supplementary Materials (Supplementary Figure S1).

### 2.11. Spheroid Formation

A total of 5 × 10^3^ cells per well were seeded into non-adherent 24-well plates to promote growth in suspension. Spheroid formation was monitored over time under standard culture conditions, and spheroid development was assessed by microscopy. Imaging was performed with the EVOS 2000 (Thermo Fisher Scientific, Waltham, MA, USA).

### 2.12. KLK5 Inhibitor Treatment

OV-MZ-6 cells stably overexpressing KLK5 and empty-vector controls were seeded in six-well plates at 300,000 cells per well and allowed to adhere for 24 h. Cells were subsequently treated with 3 µM Kallikrein 5-IN-2 (MedChemExpress; catalog no. HY-148949; Monmouth Junction, NJ, USA) or DMSO as the vehicle control for 48 h in 1 mL of complete culture medium containing 10% fetal calf serum. Following treatment, cell pellets were harvested for RNA isolation and subsequent quantification of KRT19 expression by qPCR.

### 2.13. Isolation and Cultivation of Patient-Derived Ascites Cells

Ascites was processed immediately after surgery and cultured in selection medium (Medium 199; Invitrogen) supplemented with MCDB 105 (Sigma; St. Louis, MO, USA), which allows tumor cells to survive while non-tumor cells die or fail to adhere, as described previously [45]. The cell suspension was incubated in the selection medium under standard culture conditions (37 °C, 5% CO_2_) for an initial adhesion period, allowing tumor cells to adhere while non-adherent cells were washed away. After adhesion, tumor cells were maintained in medium with FCS and PenStrep (Gibco, Thermo Fisher Scientific). Passaging was performed with 0.1% EDTA in PBS (PAN Biotech, Aidenbach, Germany).

### 2.14. Bradford Assay

Total protein concentrations in ascites samples were determined using a Bradford assay. A stock reagent containing 60 mg Coomassie Brilliant Blue G-250 in 100 mL 2 N HCl was diluted 1:1 with 2 N HCl before use. Ascites samples were diluted 1:10 and 1:25, and bovine serum albumin standards were prepared by serial dilution from a 2 mg/mL stock solution. For each measurement, 200 µL of diluted sample, standard, or water blank was mixed with 50 µL of working reagent and incubated for 30 min at room temperature. Absorbance was measured at 595 nm. Protein concentrations were calculated from the blank-corrected standard curve and adjusted for the respective sample dilution factors.

### 2.15. Enzyme-Linked Immunosorbent Assay (ELISA)

Chemokine concentrations (CXCL9) in murine ascites samples were quantified by sandwich ELISA using DuoSet ELISA kits (R&D Systems, Minneapolis, MN, USA) according to the manufacturer’s instructions. Briefly, 96-well plates were coated overnight at room temperature with capture antibodies diluted in PBS, washed with PBS containing 0.05% Tween-20, and blocked with 1× Reagent Diluent. Undiluted ascites samples and standards, prepared as seven serial 1:2 dilutions, were incubated at room temperature for 2 h. Plates were then incubated with biotinylated detection antibodies, followed by Streptavidin-HRP and TMB substrate. The reaction was stopped with 1 M sulfuric acid, and absorbance was measured at 450 nm with reference correction at 570 nm using a Multiskan FC microplate reader (Thermo Fisher Scientific, Waltham, MA, USA). Cytokine concentrations were calculated from standard curves after subtraction of the 570 nm background signal.

### 2.16. Proteomic Gene Set Enrichment Analysis (GSEA)

Cell lysis and preparation for mass spectrometry analysis were performed as described in [46]. Peptides were analyzed with the Evosep One system (Evosep Biosystems, Odense, Denmark) coupled to a timsTOF fleX mass spectrometer (Bruker) as described in [47].

Raw data were analyzed with DIA-NN software (v. 2.3.2) [48]. A spectral library was predicted using a FASTA file containing human protein sequences as of 11 March 2026 (Uniprot database). The false discovery rate (FDR) was set to 1%. The search was performed, allowing one missed cleavage, and cysteine carbamidomethylation was enabled as a fixed modification. Match between runs was enabled. Quantification was performed using the label-free quantification algorithm MaxLFQ, which calculates the protein quantities as ratios from all peptide intensities.

The data were further processed and analyzed using R (v4.3.0) in RStudio. Protein intensities were log2-transformed and median-normalized. Differential expression analysis was performed using limma [49]. *p*-values were adjusted for multiple testing using the Benjamini–Hochberg method, as implemented in limma [49].

### 2.17. Bioinformatics

RNA expression data for KLK4, KLK5, KLK6, KLK7, and KRT19 in HGSOC tissue were obtained from The Cancer Genome Atlas (TCGA) database [50] via the cBioPortal for Cancer Genomics platform (study ID: hgsoc_tcga_gdc; accessed 10 April 2026) [51,52,53]. The dataset comprised RNA sequencing data from 426 HGSOC patients. Expression values were log2-transformed prior to analysis. Data processing and statistical analyses were performed using the R programming language (version 4.5.1; R Foundation for Statistical Computing). Multivariable linear regression was performed to evaluate the independent contributions of KLK4, KLK5, KLK6, and KLK7 to KRT19 expression.

### 2.18. Software and Statistics

All experiments were conducted in at least three biological replicates. Data were analyzed and visualized using GraphPad Prism (version 11) and are presented as mean ± standard error of the mean (SEM). Quantification of cell migration in trans-well assays was performed using QuPath (version 0.5.1), followed by statistical analysis in GraphPad Prism. Kaplan–Meier estimates were performed using the log-rank test, and univariate and multivariable analyses were performed using Cox proportional hazards regression. Comparisons between two independent groups in in vitro and in vivo experiments were performed using unpaired Student’s *t*-tests. In vitro experiments included three independent biological replicates, while in vivo analyses were based on individual animals as independent biological replicates. Statistical significance was defined as *p* < 0.05. A total of 1020 proteins were identified in the experiment and used as the background for Gene Ontology over-representation analysis. Differentially abundant proteins meeting a Benjamini–Hochberg-adjusted *p*-value < 0.05 and an absolute log_2_ fold change ≥ 0.58 constituted the target list.

Two Kaplan–Meier comparisons were performed to assess the association of dichotomized KLK5 expression with overall survival and progression-free survival. For each endpoint, a multivariable Cox model included KLK5, age, ascites, and residual tumor. Survival *p*-values were nominal and were not adjusted for multiplicity; these analyses were therefore considered exploratory rather than confirmatory.

## 3. Results

### 3.1. KLK5 Is Associated with Elevated KRT19 Levels

**Figure 2:**
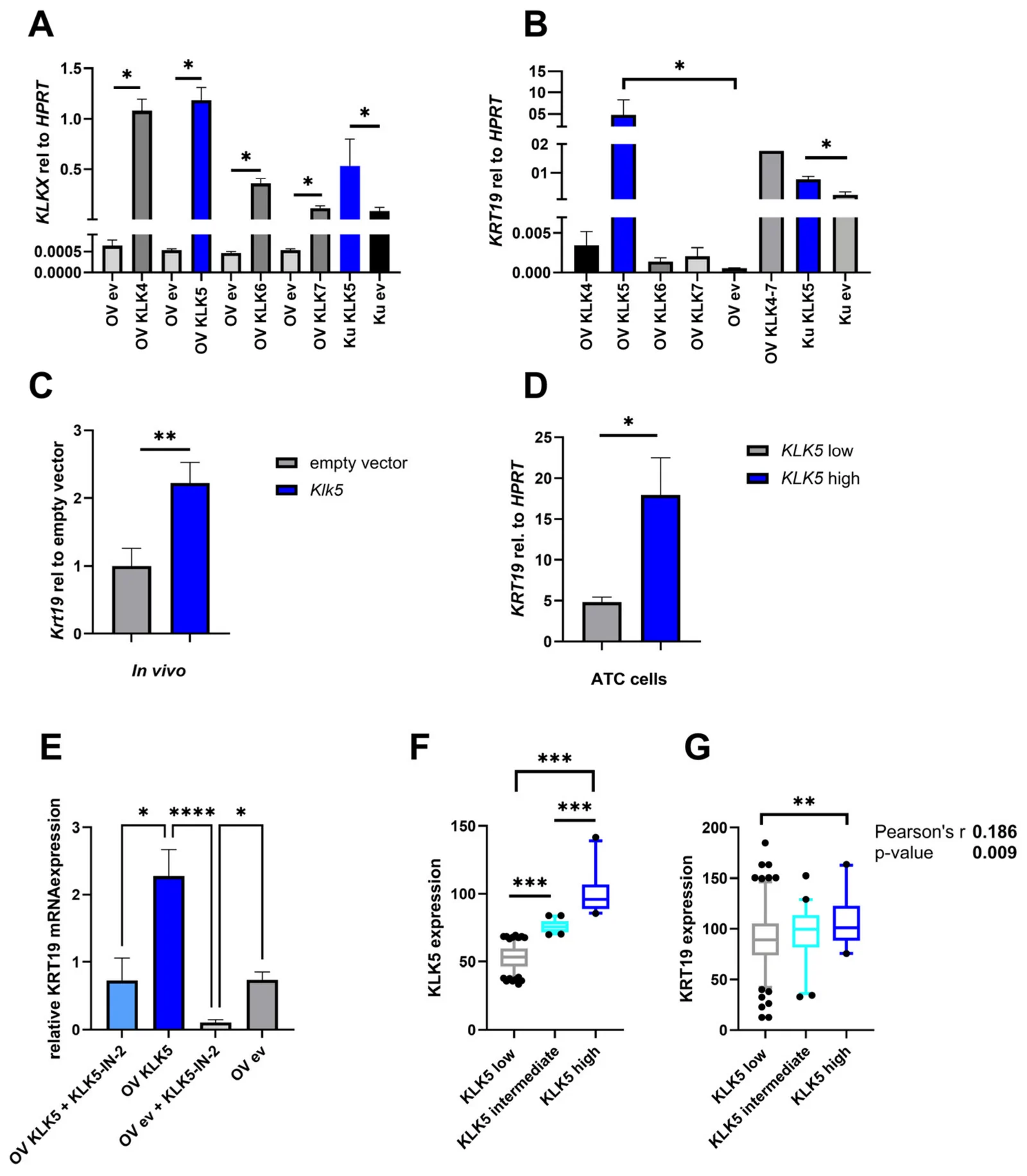
KLK-dependent regulation of KRT19 expression in vitro, in vivo, and in patient samples. (**A**) Confirmation of stable KLK overexpression of individual KLKs (KLKX) compared to empty vector controls (ev) assessed by quantitative PCR (qPCR) in OV-MZ-6 (OV) and Kuramochi (Ku) ovarian cancer cell lines (*n* = 3 biological replicates). (**B**) KRT19 expression measured by qPCR in OV-MZ-6 and Kuramochi cells in KLKX overexpressing cells, as shown in (**A**) (*n* = 3, biological rexplicates). (**C**) KRT19 expression in ID8 Trp53-/- cells stably overexpressing KLK5 compared to empty vector controls, assessed by qPCR in vivo following tumor formation and re-isolation from mice (ex vivo; empty vector: *n* = 8; KLK5: *n* = 7; each sample was derived from a different animal). (**D**) KRT19 expression in ascites-derived tumor cells (ATCs) from 6 independent patients, stratified into KLK5-high and KLK5-low groups based on median KLK5 expression (qPCR) (*n* = 6 patients were included in the analysis). (**A**–**D**) Expression levels were normalized to HPRT. (**E**) KRT19 mRNA expression analyzed by q-RT-PCR from total RNA isolated from OVMZ-6 cells with KLK5 overexpressing (OV KLK5) and empty vector control cells (OV ev). Cells were treated with 3 µM KLK5 inhibitor (KLK5-IN-2) or DMSP for 48 h (*n* = 5 biological replicates). (**F**) Immunohistochemical (IHC) analysis of KLK5 expression in a clinical HGSOC cohort (see Table S1), stratified into low, intermediate, and high expression groups (*n* = 248, matched samples for KRT19 and KLK5 epxression). (**G**) Association between KLK5 and KRT19 expression levels in the same patient cohort assessed by IHC. The correlation between KLK5 and KRT19 expression was assessed using Pearson’s correlation. All qPCR data are presented as mean ± SEM. Statistical significance was determined using unpaired *t*-tests. Statistical significance is indicated as follows: * *p* < 0.05, ** *p* < 0.01, *** *p* < 0.001; **** *p* < 0.0001.

To determine which member of the KLK4–7 cluster contributes to KRT19 regulation, we first confirmed stable overexpression of the individual KLKs 4–7 proteases in OV-MZ-6 cells and of KLK5 in Kuramochi cells by RT-qPCR (Figure 2A). Among the individually overexpressed KLKs, KLK5 was associated with the most pronounced increase in KRT19 expression in OV-MZ-6 cells. Increased KRT19 expression was also observed in cells overexpressing the KLK4–7 cluster and was independently reproduced in KLK5-overexpressing Kuramochi cells (Figure 2B).

In addition, the relationship between *Krt19* and *Klk5* was investigated in vivo by analyzing murine mesenteric tumor nodules. Following intraperitoneal injection of *Klk5*-overexpressing or empty-vector control ID8 Trp53-/- cells, tumor development was monitored, and mesenteric metastases were assessed after animal sacrifice. A highly significant increase in *Krt19* expression was observed in *Klk5*-overexpressing tumors compared to controls (Figure 2C).

To corroborate these observations in a patient-derived context, *KRT19* expression was assessed in primary ascites-derived tumor cells isolated from patients with high-grade serous ovarian cancer. Stratification based on *KLK5* expression levels (median cut-off; *n* = 6) revealed significantly higher *KRT19* expression in *KLK5*-high samples compared with *KLK5*-low samples (Figure 2D). To further investigate the functional role of KLK5 in regulating KRT19 expression, we treated OVMZ-6 cells overexpressing KLK5 and empty-vector control cells with the commercially available KLK5 inhibitor KLK5-Inh2 for 48 h. Quantitative reverse-transcription PCR (RT-qPCR) confirmed increased *KRT19* mRNA expression in KLK5-overexpressing cells. Treatment with KLK5-Inh2 reduced KRT19 mRNA expression in these cells, although this reduction did not reach statistical significance (*p* = 0.0247, Figure 2E).

Consistently, analysis of IHC data from the TUM University Hospital patient cohort (Table S1) demonstrated that KRT19 expression was significantly associated with higher KLK5 levels (r = 0.186, *p* = 0.009) (Figure 2F,G). The optimal cut-off value was determined using X-tile software (version 3.6.1.). Together, these findings support an association between KLK5 and KRT19, prompting further investigation of the three additional KLKs upregulated in HGSOC, KLK4, KLK6, and KLK7.

### 3.2. Transcriptional Association of KLKs with KRT19 in the TCGA Ovarian Cancer Cohort

To further investigate the association between *KLKs* and *KRT19* expression at the transcriptional level in a larger dataset, and to account for other KLKs frequently regulated in HGSOC, a multivariable linear regression analysis was performed using a TCGA dataset (*n* = 426; analyzed on 10 April 2026). All four *KLKs* were included simultaneously in the model to assess their independent associations with *KRT19* expression. *KLK7* (β = 0.14, *p* < 0.001) and KLK5 (β = 0.06, *p* = 0.010) remained significantly associated with *KRT19* expression, whereas *KLK6* and *KLK4* were not. Variance inflation factor (VIF) analysis indicated no evidence of relevant multicollinearity among the predictors (all VIFs < 2). The overall model explained approximately 13% of the variance in KRT19 expression (R^2^ = 0.13) (Table 1). These findings indicate that *KLK5* and *KLK7* are independently associated with KRT19 expression at the transcriptional level.

### 3.3. Functional Impact of KLK5 in Ovarian Cancer Cells and on Immune-Related Pathways

**Figure 3:**
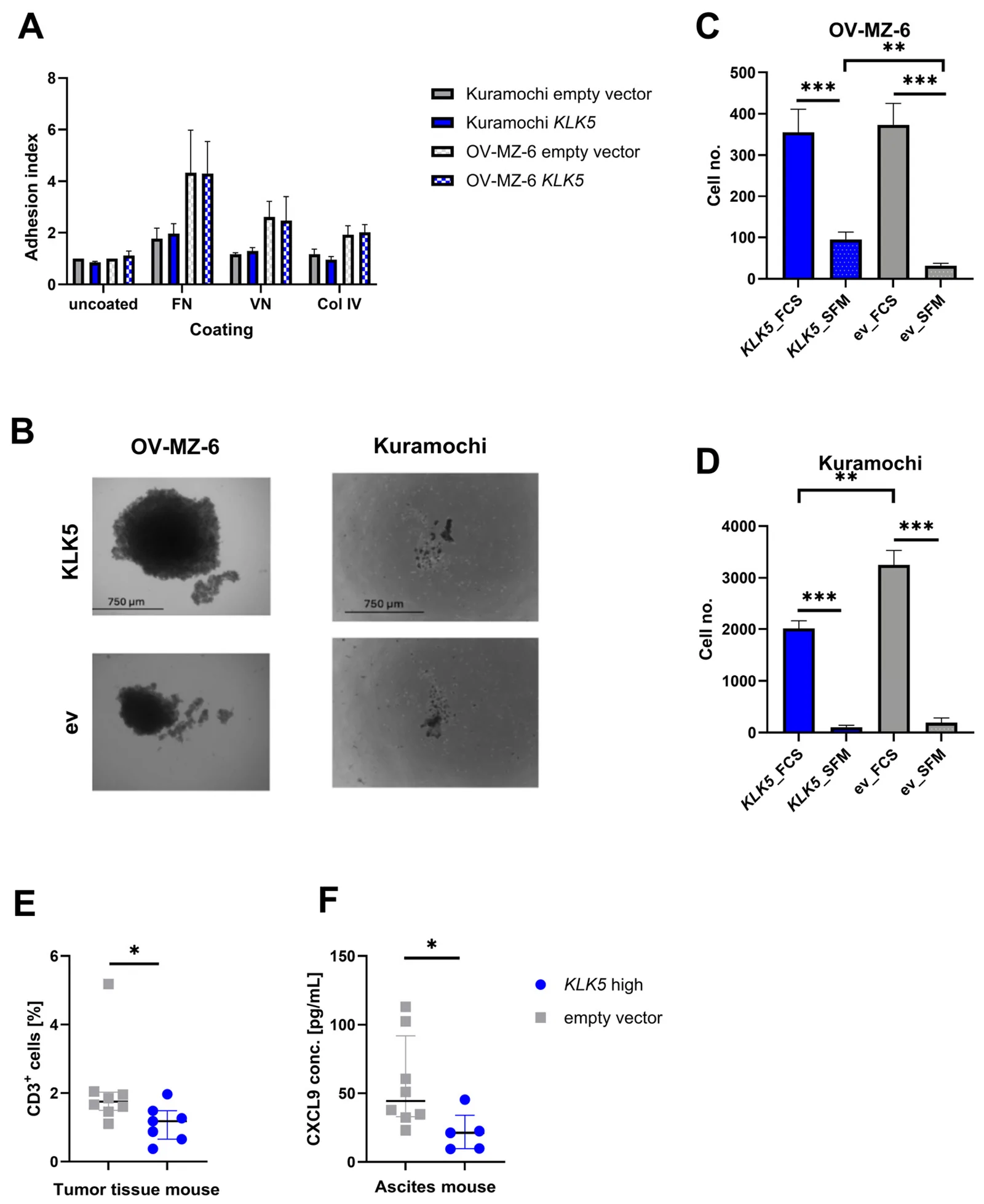
KLK5 modulates cell adhesion, spheroid formation, migration, and chemokine secretion in ovarian cancer models. (**A**) Cell adhesion assay (Landgren method) performed in Kuramochi and OV-MZ-6 ovarian cancer cells stably overexpressing KLK5 compared to empty vector (ev) controls (*n* = 3). Adhesion to extracellular matrix (ECM) components, including fibronectin (FN), vitronectin (VN), and collagen IV (Col IV), was assessed. The fold change was calculated relative to the adhesion of the empty vector in the uncoated condition. (**B**) Representative images of spheroid formation capacity in OV-MZ-6 and Kuramochi cells with KLK5 overexpression or empty vector control after 10 days in culture. (**C**,**D**) Migration capacity was assessed by trans-well assays in OV-MZ-6 (**C**) and Kuramochi (**D**) cells, comparing KLK5-overexpressing cells to empty vector controls (*n* = 3). Directed migration was induced by 20% FCS as a chemoattractant, while undirected migration was assessed under serum-free (SFM) (starvation) conditions. (**E**) Percentage of CD3+ cells of total cells assessed via immunohistochemical staining of murine tumor tissue (Klk5 high *n* = 7 vs. empty vector *n* = 8). (**F**) CXCL9 concentrations in mouse ascites measured by ELISA comparing Klk5-high tumors and empty vector controls. CXCL9 values have been normalized to total protein content in ascitic fluid as determined by Bradford assay (Klk5 high *n* = 5 vs. empty vector *n* = 8, samples taken from individual animals). (**E**,**F**) Each dot represents one biological replicate, with the total number of dots corresponding to the sample size (*n*). Data are presented as mean ± SEM. Statistical significance was determined using unpaired *t*-tests. Statistical significance is indicated as follows: * *p* < 0.05, ** *p* < 0.01, *** *p* < 0.001.

To investigate the effects of KLK5 on tumor-related cellular functions, we performed a series of in vitro assays (Figure 3A–D) as well as in vivo experiments (Figure 3E,F).

Analysis of KLK5-mediated effects on cell adhesion to ECM proteins did not reveal significant differences in adhesion capacity between *KLK5*-overexpressing and control cells under any of the conditions tested (Figure 3A). Notably, OV-MZ-6 cells exhibited generally stronger adhesion to fibronectin (FN) and vitronectin (VN) than Kuramochi cells, regardless of KLK5 expression. KLK5 overexpression markedly enhanced spheroid formation in OV-MZ-6 cells compared with empty-vector controls, as demonstrated by representative images after 10 days of culture (Figure 3B, Supplementary Figure S1). To exclude potential proliferation-related effects, a proliferation assay was performed, demonstrating that KLK5 overexpression does not affect cell proliferation in the human cell model (Supplementary Figure S2). In contrast, Kuramochi cells showed no evident spheroid formation under either condition. In trans-well migration assays, *KLK5* overexpression significantly increased undirected migration in OV-MZ-6 cells (Figure 3C), while no significant effect on directed migration toward 20% FCS was observed. In Kuramochi cells, no differences in undirected migration were detected; however, directed migration was significantly higher in empty-vector control cells than in *KLK5*-overexpressing cells (Figure 3D). Migration was quantified as the number of cells per field (Supplementary Figure S3).

Finally, the potential impact of KLK5 on immune-related pathways was assessed by analyzing T-cell abundance in mouse tumor tissue (*Klk5-high* vs. empty-vector mice) using CD3 staining, which revealed a significant decrease in CD3+ cells in *Klk5*-overexpressing tumors (Figure 3E). Accordingly, levels of the T-cell-recruiting chemokine CXCL9 were assessed in the corresponding mouse ascites. CXCL9 levels were significantly decreased in *Klk5*-overexpressing tumors compared with the empty vector control (Figure 3F).

### 3.4. Proteomic Profiling Identifies Cholesterol Homeostasis as a Major KLK5-Associated Program

**Figure 4:**
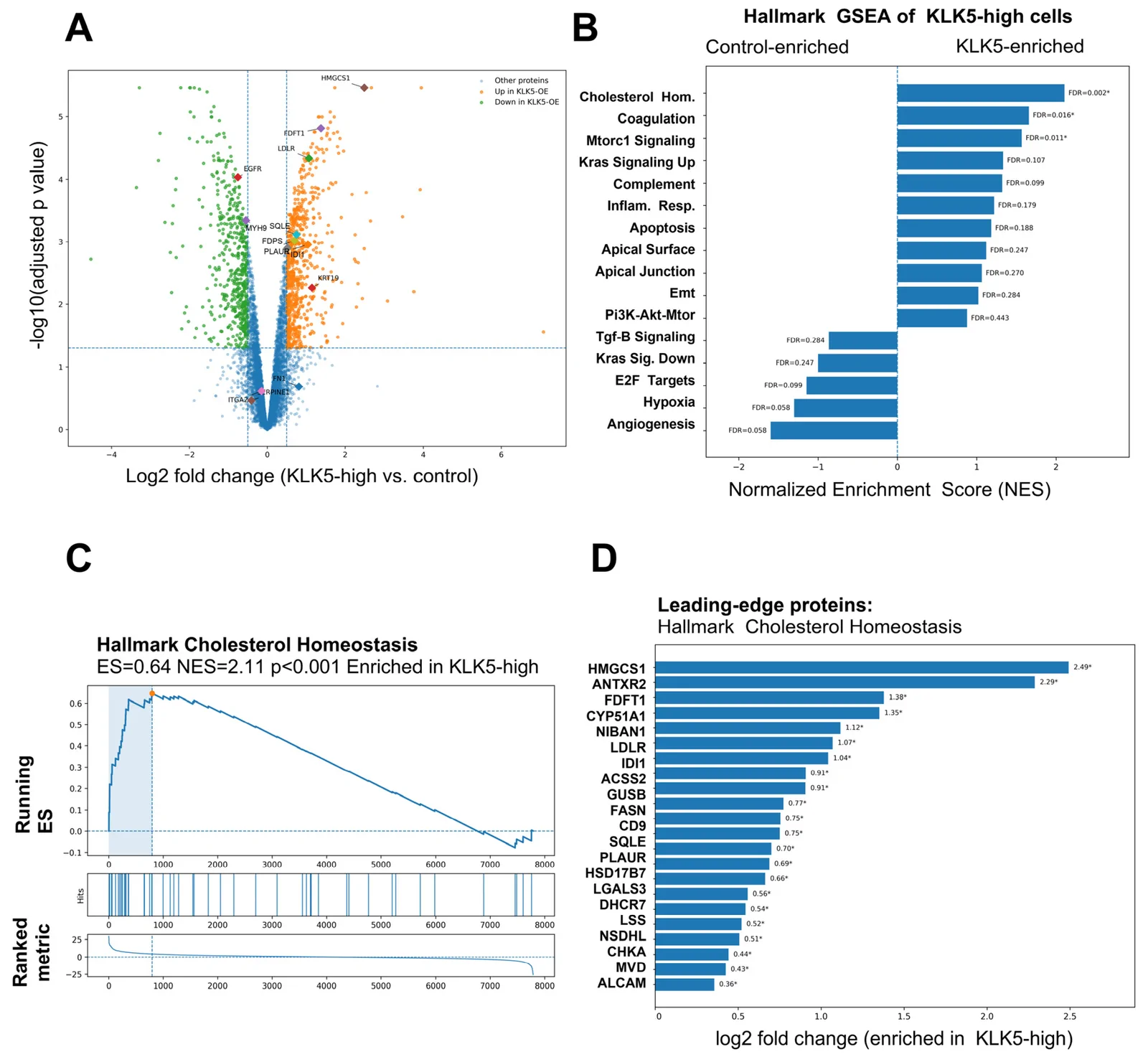
Proteomic profiling identifies cholesterol homeostasis as the dominant KLK5-associated program. (**A**) Volcano plot of protein abundance in KLK5-overexpressing versus empty-vector OV-MZ-6 cells. The *x*-axis shows log_2_ fold change, and the *y*-axis shows log_10_ (adjusted *p* value). Proteins meeting the indicated fold-change and adjusted-*p*-value thresholds are shown as increased (orange) or decreased (green) in KLK5-overexpressing cells; selected proteins are labeled. (**B**) Hallmark gene set enrichment analysis of the ranked proteomic dataset. Positive normalized enrichment scores (NES) indicate enrichment in KLK5-overexpressing cells and negative scores indicate enrichment in control cells. FDR values are shown; * FDR < 0.05. (**C**) Enrichment plot for HALLMARK_CHOLESTEROL_HOMEOSTASIS (enrichment score [ES] = 0.64; NES = 2.11; nominal *p* = 9.999 × 10^−5^), enriched toward the KLK5-high end of the ranked protein list. (**D**) Log_2_ fold changes of leading-edge proteins contributing to cholesterol-homeostasis enrichment. * Adjusted *p* < 0.05.

To identify additional pathways associated with KLK5 overexpression, we performed quantitative proteomic profiling of KLK5-overexpressing and empty-vector OV-MZ-6 cells. Differential abundance analysis identified a broad set of significantly altered proteins, including increased KRT19 and pronounced upregulation of several cholesterol- and mevalonate-pathway proteins, such as HMGCS1, FDFT1, and LDLR (Figure 4A).

Ranked Hallmark gene set enrichment analysis identified cholesterol homeostasis as the strongest KLK5-enriched program (normalized enrichment score (NES) = 2.11, FDR = 0.002), followed by coagulation (FDR = 0.016) and mTORC1 signaling (FDR = 0.011; Figure 4B). None of the other displayed Hallmark gene sets reached the predefined significance threshold of FDR < 0.05. The cholesterol-homeostasis enrichment curve showed concentration of pathway members at the KLK5-high end of the ranked protein list (Figure 4C). Leading-edge proteins included HMGCS1, FDFT1, CYP51A1, LDLR, IDI1, ACSS2, FASN, SQLE, HSD17B7, DHCR7, LSS, FDPS, NSDHL, and MVD, together with additional proteins involved in membrane organization and tumor-cell behavior (Figure 4D). These data reveal coordinated remodeling of sterol and lipid-associated pathways rather than an isolated change in individual proteins.

### 3.5. KLK5 Expression Is Associated with Impaired Survival in Patients and In Vivo

**Figure 5:**
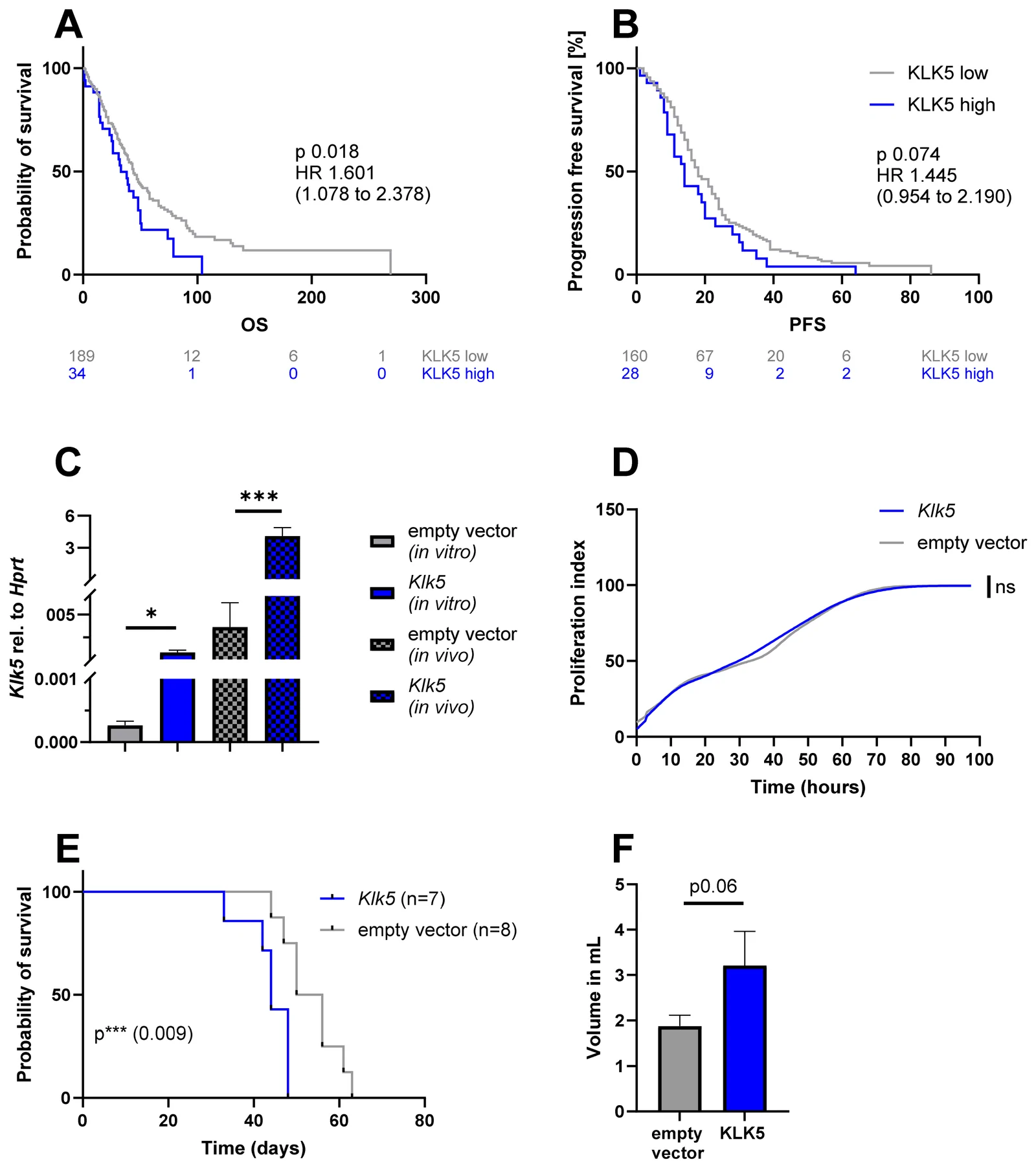
Clinical and in vivo associations of KLK5 with survival in ovarian cancer. (**A**,**B**) Kaplan–Meier analysis of overall survival (**A**) and progression-free survival (**B**) in an internal cohort of high-grade serous ovarian cancer (HGSOC) patients (details in Table S1), collected between 1990 and 2014. Patients were stratified into KLK5 high and low-expressing groups based on an optimal cut-off determined by X-tile analysis (84th percentile). Survival differences were assessed using the log-rank test. Hazard ratios (HR) were calculated from log-rank analysis (reciprocal) using GraphPad Prism. (**C**) Klk5 expression in ID8 Trp53-/- cells was measured by quantitative PCR (qPCR), normalized to the housekeeping gene Hprt. Expression was assessed in vitro prior to injection (*n* = 3) and in vivo after tumor growth and re-isolation from tumor tissue (empty vector: *n* = 8; KLK5: *n* = 7). Statistical significance was determined using the unpaired *t*-test. (**D**) Proliferation of ID8 Trp53-/- cells assessed using the Cellwatcher PHIO live-cell imaging system (*n* = 3). (**E**) Kaplan–Meier survival analysis of C57BL/6JRj mice intraperitoneally injected with ID8 Trp53-/- cells (empty vector: *n* = 8; KLK5: *n* = 7). Survival differences were analyzed using the log-rank test. (**F**) Ascites volume at endpoint in mice injected with ID8 Trp53-/- cells (empty vector: *n* = 8; KLK5: *n* = 7). Data are presented as mean ± SEM. Statistical analysis was performed using an unpaired *t*-test. Statistical significance is indicated as follows: * *p* < 0.05, *** *p* < 0.001, ns: not significant.

To examine the clinical relevance of KLK5 in HGSOC, the overall impact of KLK5 in ovarian cancer was assessed in a well-characterized HGSOC patient cohort (Table S1) and also in an in vivo mouse model. Kaplan–Meier survival analysis of the TUM University Hospital patient cohort revealed that elevated KLK5 expression was significantly associated with reduced overall survival (nominal *p* = 0.018; Figure 5A), whereas the association with reduced progression-free survival did not reach statistical significance (nominal *p* = 0.074; Figure 5B). Multivariable analyses, including age, FIGO stage, and residual tumor burden, did not reveal a significant association between KLK5 expression and either overall survival or progression-free survival in the same cohorts (Table 2). Moreover, KLK5 did not show a statistically significant association with overall survival as a continuous variable (HR 1.002 (0.994–1.011), *p* = 0.581). Residual tumor burden emerged as an independent predictor of overall survival. For progression-free survival, ascitic fluid, residual tumor, and age were significant predictors.

To functionally analyze these clinical observations in vivo, a syngeneic mouse model was employed. ID8 Trp53-/- cells stably overexpressing *Klk*5 (Figure 5C) did not display increased proliferative capacity compared with empty vector control cells in vitro (Figure 5D) and were subsequently inoculated intraperitoneally. Stable overexpression was confirmed, and increased *Klk5* expression was generally detectable in vivo in both the empty vector and *Klk5* groups compared with in vitro conditions prior to inoculation (Figure 5C). Mice bearing *Klk5*-high tumors exhibited significantly reduced survival compared with control animals and reached predefined humane endpoints earlier (Figure 5E). Furthermore, *Klk5*-high mice developed markedly increased ascites volumes (Figure 5F). Because tumors from Klk5-overexpressing mice showed significantly higher Krt19 expression (Figure 2C), the animals with reduced overall survival and increased ascites were also those with higher Krt19 levels.

## 4. Discussion

**Figure 6:**
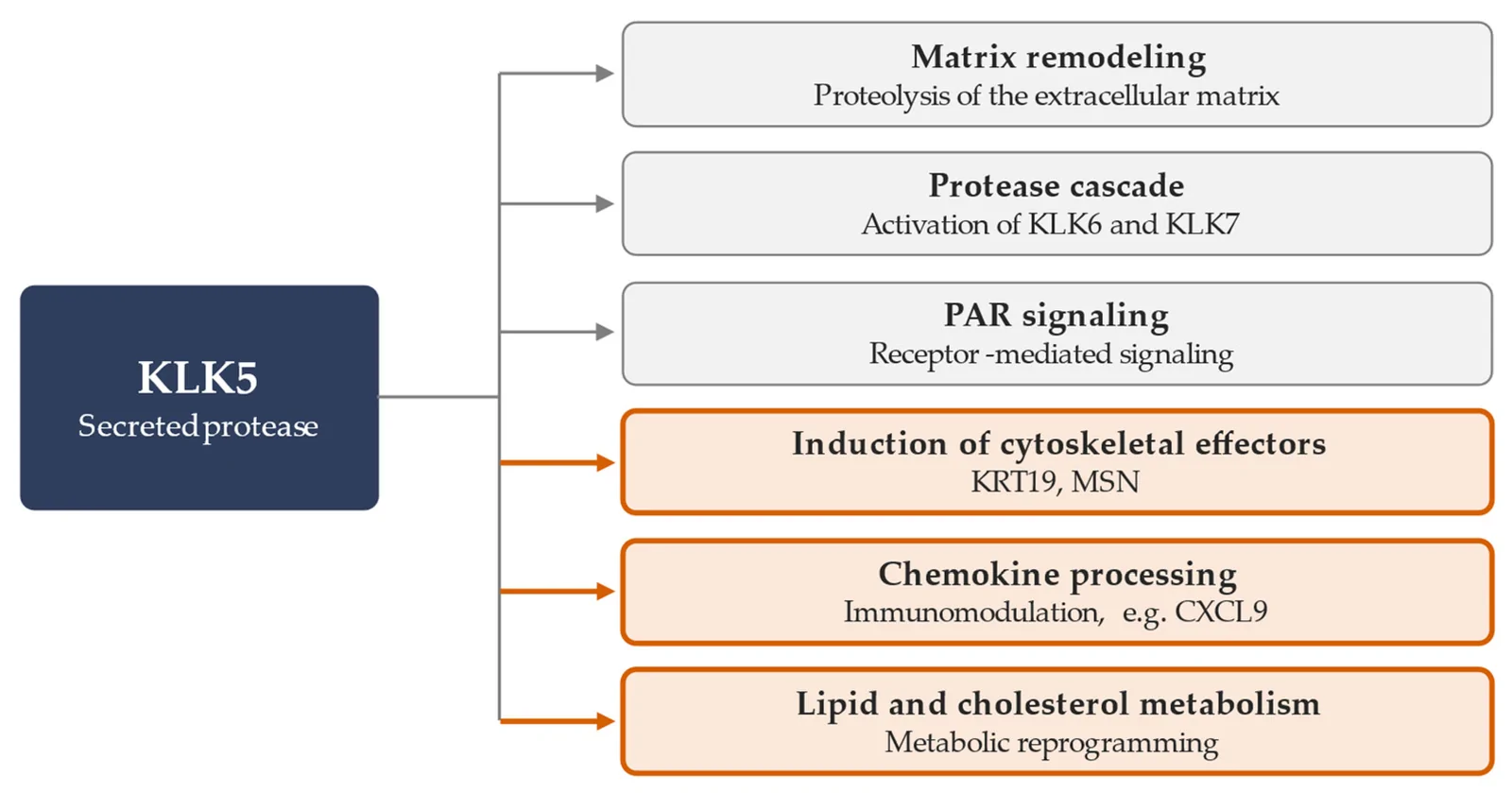
Proposed mechanisms by which KLK5 may affect tumor biology. Highlighted pathways were experimentally analyzed and shown to promote tumor progression.

As a secreted serine protease with a broad substrate repertoire, KLK5 may influence tumor progression through several mechanistically distinct effects rather than through a single linear pathway. In this study, we delineated the KLK5-dependent tumor biology of HGSOC across tumor cell behavior, the cellular proteome, the ascitic immune milieu, and in vivo disease progression, and identified three tumor-relevant programs (Figure 6). First, the connection to KRT19 expression was drawn from the previously established KLK4–7/KRT19 association [13] and from our observation that, among KLK4–7, KLK5 alone upregulated KRT19 when overexpressed individually. Second, a reduction in the CXCR3 ligand CXCL9, accompanied by diminished CD3+ cell abundance, was observed in vivo, emerging from the ascitic microenvironment of tumor-bearing mice. Third, a coordinated remodeling of cholesterol/mevalonate metabolism, coupled to mTORC1 signaling, was unbiased and data-driven, arising not from a prior hypothesis but from the most strongly enriched Hallmark program in unbiased proteomic profiling. In the following, the 3 axes of KLK5-driven tumor progression will be discussed separately.

### 4.1. KLK5 and KRT19

KRT19 has been implicated in tumor progression and chemoresistance across multiple cancer types, and recent evidence specifically supports its role as a prognostic marker and contributor to metastasis and paclitaxel resistance in HGSOC [30,54]. Wang et al. previously demonstrated that the combined overexpression of KLK4–7 regulates KRT19 at both the mRNA and protein levels in serous ovarian cancer cells, with significant positive associations for KRT19/KLK4 and KRT19/KLK5 at the protein level, as observed by immunohistochemistry [13].

For in vitro analyses, the cell line models Kuramochi and OV-MZ-6 were selected. Kuramochi cells received the highest suitability score as an HGSOC model in the seminal cross-comparison of ovarian cancer cell lines with TCGA tumors, closely recapitulating the copy-number landscape, mutation frequency, and key genetic lesions of patient-derived HGSOC (including TP53 and BRCA2 mutation and functional homologous-recombination deficiency), in contrast to historically popular but molecularly atypical lines such as SKOV3 and A2780 [55,56,57,58]. Although OV-MZ-6 has not been formally ranked in panels assessing genomic fidelity, it is an established serous, ascites-derived ovarian carcinoma cell line that provides continuity with previous work [13].

In vitro experiments demonstrated that among the four individually overexpressed KLKs (KLK4–7), only KLK5-overexpressing cells showed a significant increase in KRT19 expression. Additionally, KLK5 overexpression was confirmed at the protein level in OV-MZ-6 cells (Supplementary Figure S4). This finding was reproducible across an additional cell line and corroborated in vivo, suggesting that the tumor-relevant effects of KLK5 overexpression may involve KRT19 upregulation. An exploratory analysis of ATCs from six patients also revealed increased KRT19 expression in the three patients exhibiting higher KLK5 expression. However, given the limited sample size, these findings should be interpreted with caution and regarded as preliminary supportive evidence rather than definitive proof of an association between KLK5 and KRT19 expression. The significant reduction in KRT19 mRNA expression following KLK5 inhibitor treatment (*p* = 0.0247) supports a contribution of KLK5 proteolytic activity to KRT19 regulation in KLK5-overexpressing OVMZ-6 cells. These findings suggest that the observed increase in KRT19 expression may depend, at least in part, on KLK5 enzymatic activity. However, pharmacological inhibition alone does not establish the specificity or underlying mechanism of this relationship. Complementary genetic approaches and assessment of KRT19 protein expression would further strengthen this interpretation. Consistent with this, we observed a significant positive association between KLK5 and KRT19 protein expression in our institutional (TUM) patient cohort by immunohistochemistry.

A notable limitation of this analysis is that, except for the initial in vitro screen, the other KLKs were not simultaneously assessed as potential regulators or interacting factors of KRT19. To address this, a multivariate analysis of a TCGA dataset was performed, and KLK7 was identified as a potential additional regulator of the potential *KLK5-KRT19* axis. This hypothesis is biologically plausible for several reasons. First, KLK5 and KLK7 are the two major proteases involved in epidermal desquamation and function in a coordinated proteolytic cascade, in which KLK5 may serve as the physiological activator of KLK7 [59,60]. Second, concordant co-expression of KLK5 and KLK7 has been demonstrated in ovarian cancer at both the mRNA and protein levels [20,59]. Third, Gong et al. [20] reported significant positive correlations between KLK5 and KLK7 in HGSOC, with KLK7 independently associated with unfavorable prognosis for both progression-free and overall survival in multivariate analysis, exhibiting even stronger predictive value than KLK5 alone. However, it must be acknowledged that the current data do not establish a direct mechanistic link between KLK5 and KRT19 regulation, even though they strongly support an association between KRT19 and KLK5.

### 4.2. Functional Impact of KLK5 on Ovarian Cancer Cell Behavior

If KLK5 were to exert its tumor-promoting effects primarily by upregulating KRT19, one would expect KLK5-overexpressing cells to phenocopy the functional profile of KRT19-overexpressing cells. We previously found that KRT19 promotes adhesion to ECM proteins, enhances intercellular adhesion by stabilizing E-cadherin at the cell surface, and increases directed migration in ovarian cancer cells [30,61,62]. Additionally, KRT19 promotes spheroid formation and integrity, processes considered critical for transcoelomic dissemination of ovarian cancer [30,61].

In our experiments, *KLK5*-overexpressing OV-MZ-6 cells exhibited enhanced spheroid formation compared to empty-vector controls, consistent with the known effects of KRT19 on spheroid integrity [30]. This finding also aligns with previous reports demonstrating that combined KLK4–7 overexpression promotes spheroid growth in three-dimensional culture systems, particularly in the presence of integrin-engaging RGD motifs [63]. However, *KLK5* overexpression did not recapitulate the full spectrum of KRT19-mediated functional effects. Specifically, no KLK5-mediated enhancement of adhesion to ECM molecules was observed. This is noteworthy, as KRT19 has been shown to promote adhesion to ECM substrates in ovarian cancer cells [30].

Similarly, directed migration, which is significantly enhanced by KRT19 overexpression, was not consistently increased by KLK5 overexpression across both cell lines tested [30,62]. This discrepancy further suggests that the functional relationship between KLK5 and KRT19 is not a simple linear pathway, but rather that KLK5 modulates a broader set of downstream effectors, potentially with opposing effects on migration. Indeed, KLK5 has been shown to activate proteinase-activated receptors (PARs) and to process ECM components, growth factors, and other proteases, all of which could independently influence migratory behavior in directions that partially offset KRT19-mediated pro-migratory signals [8,9,64].

The most plausible reason for the divergent effects is the opposite baseline state of the two lines. OV-MZ-6 expresses almost no endogenous KLK4–7 and is more adhesive and less motile than Kuramochi cells; consequently, the introduced KLK5 acts as a de-adhesion/activation switch, increasing undirected (random) migration [13,14,40]. Kuramochi, by contrast, already exhibits high motility and a hybrid-mesenchymal phenotype, leaving limited scope for further de-adhesion. Additional KLK5 may therefore perturb directional migration capacities [56,57]. In OV-MZ-6, the KLK network may promote migration by activating TGFβ-1 signaling, whereas in the already highly oncogenic Kuramochi cells, KLK5-induced activation of the RhoA/NF-κB pathways may be redundant or counterproductive, thereby disrupting front–rear polarity [12,13,40,55]. Such context-dependent, sometimes biphasic effects are well described for the KLK family [9,65]. This remains a mechanistic hypothesis, however, as no direct head-to-head comparison between these two lines has been reported in the literature to date.

Taken together, the selective overlap between KLK5- and KRT19-mediated phenotypes, with concordance in spheroid formation but discordance in ECM adhesion and directed migration, suggests that KRT19 upregulation represents one component of a multifaceted KLK5-driven program, rather than the sole mediator of KLK5’s tumor-promoting effects. Additional KLK5 substrates and signaling pathways likely contribute to the net functional outcome in a context-dependent manner.

The fact that KRT19 effects on tumor behavior are not fully recapitulated by KLK5 suggests that the proposed KLK5–KRT19 axis is not the predominant pathway mediating KLK5’s effects on tumor biology. Rather, they appear to result from the interplay of several KLK5-regulated processes, with modulation of KRT19 representing one component of a broader regulatory network.

### 4.3. KLK5-Mediated Modulation of the Chemokine Microenvironment

Beyond the KRT19-associated phenotypes discussed above, we observed that KLK5 may influence immune-related processes and thereby affect tumor-promoting effects. Importantly, we found that the proportion of CD3+ cells was significantly reduced in the Klk5-overexpressing group, as were CXCL9 concentrations. Based on these observations, we propose that KLK5 may contribute to the establishment of an immunosuppressive peritoneal microenvironment by reducing the availability of CXCL9 and thereby impairing T-cell recruitment. However, whether KLK5 directly mediates CXCL9 proteolysis or indirectly influences its expression through alterations in inflammatory signaling remains unresolved. Furthermore, a causal relationship between reduced CXCL9 levels and impaired T-cell recruitment has not yet been established. Nevertheless, this finding carries important clinical implications, as CXCL9 is one of the most robust favorable prognostic factors in HGSOC. Bronger et al. [35] demonstrated that high intratumoral CXCL9 expression was associated with approximately a twofold increase in overall survival in two independent HGSOC cohorts, and that CXCL9 levels correlated with tumor-infiltrating CD3+ T lymphocytes. Additionally, Seitz et al. [37] showed that CXCL9 overexpression was sufficient to drive T-cell accumulation, delay ascites formation, and enable successful anti-PD-L1 therapy in an otherwise immune checkpoint blockade-resistant ovarian cancer model. The proposed KLK5-mediated reduction in CXCL9 could therefore represent a previously unrecognized mechanism of immune evasion in HGSOC.

The selective reduction in CXCL9 is noteworthy, given its important role in antitumor immune responses. CXCL9 is predominantly IFNγ-dependent and primarily macrophage-derived [36,66] and contributes to the recruitment of CXCR3-expressing effector immune cells into the tumor microenvironment. Thus, the reduction in CXCL9 observed upon KLK5 overexpression may contribute to impaired immune cell recruitment and shift the tumor microenvironment toward immunosuppression. This interpretation is consistent with the reduced CD3+ T-cell infiltration observed in KLK5-overexpressing tumors.

Proteolytic inactivation of chemokines is a recognized immune-escape mechanism, and matrix metalloproteases have been shown to cleave CXCL9 at its C-terminus, thereby reducing chemotactic activity [67,68]. Given KLK5’s established capacity to proteolytically process chemokines such as CCL14, direct cleavage of CXCL9 is a plausible mechanism warranting further investigation [8]. Alternatively, KLK5 could indirectly suppress CXCL9 by modulating IFNγ signaling or macrophage polarization [36].

Regardless of the mechanism, KLK5-mediated reduction in CXCL9 could add an immunomodulatory dimension to its tumor-promoting effects, complementing its roles in ECM remodeling and KRT19-mediated metastasis. This also raises the possibility that KLK5-high tumors might be less responsive to immune checkpoint blockade [37]. This finding suggests that blocking KLK5 with an antibody may enhance the efficacy of immune checkpoint blockade.

### 4.4. Proteomic Evidence Links KLK5 to Cholesterol Homeostasis and mTORC1 Signaling

To identify KRT19-independent components of the KLK5 phenotype, we performed unbiased proteomic profiling of KLK5-overexpressing and control OV-MZ-6 cells. Cholesterol homeostasis emerged as the most strongly enriched Hallmark gene set (NES = 2.11; FDR = 0.002), driven not by a single outlier but by coordinated increases across sterol-metabolic (HMGCS1, FDFT1, CYP51A1, SQLE, LSS, MVD, FDPS, DHCR7, NSDHL, HSD17B7, and LDLR), with HMGCS1 and FDFT1 among the most strongly upregulated individual proteins. This broad remodeling of the cholesterol/mevalonate machinery provides a plausible additional explanation for why the KLK5 phenotype only partially overlapped with that of KRT19 alone. The direction of the present cholesterol/mevalonate signature is opposite to that reported in MDA-MB-231 breast cancer cells, where re-expression of KLK5 in an otherwise KLK5-negative background repressed the mevalonate pathway and suppressed malignancy [15]. This metabolic divergence mirrors the divergent functional roles of KLK5 across tumor entities: whereas KLK5 can act in a tumor-suppressive manner in breast cancer, it is consistently overexpressed and associated with more aggressive behavior in HGSOC, cervical, and colorectal carcinoma [12,15,69,70]. Rather than indicating a universal metabolic function of KLK5, this contrast is consistent with the broadly context-dependent and at times opposing roles described for KLKs across tumor entities, which are shaped by the available substrate repertoire, endogenous inhibitors, receptor context, and whether the enzyme is re-expressed in a naive background or overexpressed within a permissive oncogenic circuitry [9].

The parallel enrichment of mTORC1 signaling (FDR = 0.011) offers a biologically coherent link, as SIK2 and MIEF2 activate SREBP-dependent lipid synthesis via PI3K/AKT or AKT/mTOR signaling in ovarian cancer models [71,72] and the mTOR effector p70S6K promotes spheroid implantation and peritoneal dissemination through P-cadherin/β1-integrin signaling [73]. However, the mTORC1 Hallmark signal should not be equated with direct mTORC1 activation, and confirmation will require analysis of phospho-S6K, phospho-S6, phospho-4EBP1, and SREBP2 processing.

The ovarian cancer context supports the potential relevance of this response: ascitic cholesterol activates LXRα/β, induces MDR1, and promotes cisplatin resistance, with higher levels linked to shorter recurrence-free survival [74]. Furthermore, activation of the mevalonate pathway accompanies progression in TP53-mutant murine oviductal models and confers sensitivity to simvastatin [75]. In addition, statins and zoledronic acid reduce ovarian cancer cell viability primarily through impaired geranylgeranylation [76]. Because cholesterol and non-sterol isoprenoids support membrane organization, small GTPase prenylation, and stress adaptation, the KLK5-associated program could facilitate the survival and cohesion of detached tumor-cell aggregates. Still, this remains a mechanistic inference, as the proteomic data quantify protein abundance rather than sterol flux, cholesterol content, or prenylation.

Coagulation was the third significantly enriched Hallmark program (FDR = 0.016), whereas complement, inflammatory response, epithelial–mesenchymal transition (EMT), apical junction, and PI3K–AKT–mTOR showed only non-significant directional enrichment. This distinction is important since the proteome supports a focused cholesterol/mTORC1/coagulation-associated state rather than a generalized EMT or inflammatory program. Upregulation of the urokinase-type plasminogen activator receptor (uPAR; PLAUR) within the leading-edge cholesterol signature is potentially relevant, as malignant ascites can activate the uPA/uPAR system in invasive ovarian cancer cells, thereby enhancing Ras/ERK signaling and invasion [77]. Conversely, the lack of significant EMT enrichment is compatible with the functional data, in which KLK5 enhanced spheroid formation without consistently increasing matrix adhesion or directed migration. Together with a recent single-cell and spatial analysis linking KLK5-high HGSOC epithelial cells to stromal remodeling and an immunosuppressive microenvironment [69], the present findings position KLK5 as an upstream organizer of several context-dependent tumor programs rather than as a linear inducer of KRT19 or canonical EMT.

This metabolic dimension may also intersect with the immune phenotype. Recent analysis of HGSOC ascites showed that uptake of polar lipids, particularly phosphatidylcholine, disrupts NK-cell membrane organization and cytotoxicity [78]. Whether KLK5-dependent metabolic remodeling contributes to both tumor-cell fitness and immune dysfunction warrants studies quantifying cellular and ascitic sterols and phospholipids alongside NK/T-cell function, CXCL9 abundance, and tumor progression. If validated, a KLK5–mTORC1–SREBP/mevalonate axis would broaden the translational implications of KLK5 and rationalize combining KLK5 inhibition with statins, prenylation-directed agents, or mTOR-pathway inhibitors, though such combinations remain hypothesis-generating, as neither metabolic flux nor therapeutic dependence was tested here.

### 4.5. Impact of KLK5 on Prognosis in HGSOC

Our clinical findings support a prognostic role for KLK5 in ovarian cancer. High KLK5 expression was significantly associated with shorter overall survival and showed a trend toward shorter progression-free survival in the Kaplan–Meier analysis, although it did not emerge as an independent prognostic factor in the multivariate analysis. These results are broadly consistent with previous studies linking elevated KLK5 expression to aggressive clinicopathological characteristics and unfavorable outcomes in ovarian cancer [79,80,81]. In the seminal study by Kim et al. [79], KLK5 expression was detected in 41% of ovarian cancer specimens and was associated with an increased risk of relapse and death in univariate analysis. Gong et al. [82] and Diamandis et al. [80] likewise reported prognostic associations for KLK5, although its independent predictive value was attenuated in multivariate analyses that included established clinical factors such as residual tumor burden. Thus, while KLK5 may not provide prognostic information entirely independent of established clinicopathological parameters, the significant association with overall survival observed in our cohort supports its relevance as a marker of biologically aggressive disease.

This clinical association was complemented by our in vivo findings using the ID8 Trp53-/- cell line. The parental ID8 model is limited by its ovarian surface epithelial origin and Trp53 wild-type status, whereas TP53 alterations are nearly universal in human HGSOC. CRISPR-mediated Trp53 deletion addresses this limitation, and ID8 Trp53-/- is therefore an established immunocompetent model for studying ascites formation and the tumor microenvironment [55,83,84,85]. Pronounced KLK5 overexpression significantly impaired survival in the mouse model and was accompanied by a trend toward increased ascites formation, providing functional evidence that KLK5 can promote ovarian cancer progression. These observations are consistent with the xenograft study by Pépin et al. [65], in which KLK5 overexpression in ES-2 ovarian cancer cells altered tumor pathophysiology. Notably, however, the effects of KLK5 in that model depended on its co-expression with other KLKs, including KLK6 and KLK10, highlighting the importance of the broader protease network and cellular context. Interestingly, although KLK5 overexpression remained elevated in mouse tumors derived from KLK5-overexpressing cells compared with empty-vector controls, KLK5 expression was markedly increased in both groups compared with the corresponding in vitro conditions. Such a pronounced increase, especially of proteases, is not uncommon in in vivo tumor models and may reflect the substantial influence of the tumor microenvironment on gene regulation [86,87]. In particular, interactions with stromal and immune cells, as well as changes in extracellular matrix composition, inflammatory signaling, and hormonal or other host-derived stimuli, may induce endogenous KLK5 expression in tumor cells [9,69,80,88,89]. In addition, the analyzed tumor tissue contains not only tumor cells but also cells of the surrounding tumor microenvironment, which may contribute to the overall KLK5 signal. Thus, the markedly increased KLK5 expression observed in vivo may reflect a combination of microenvironmental induction of endogenous KLK5 and contributions from non-tumor cells, highlighting that KLK5 regulation in vivo is considerably more complex than in the isolated in vitro setting. Taken together, the concordant clinical and experimental findings indicate that KLK5 is not merely associated with advanced ovarian cancer but may actively contribute to disease progression and reduced survival. Nevertheless, its precise contribution is likely shaped by tumor context, interactions with other KLKs, and established clinical determinants of outcome.

## 5. Limitations

Several limitations of the present study should be acknowledged. First, although the correlation between KLK5 and KRT19 expression in the clinical cohort was statistically significant (*p* = 0.009), it was weak (r = 0.186), suggesting that their relationship in vivo is likely influenced by complex regulatory networks and other biological factors. Similarly, the analysis of primary ATCs was based on a relatively small sample size (*n* = 6) and, therefore, warrants confirmation in larger patient-derived cohorts. Furthermore, the present study provides evidence for an association between KLK5 and KRT19 and supports a potential KLK5–KRT19 axis, but does not establish a direct mechanistic relationship between the two proteins. In particular, complementary loss-of-function or will be required to determine whether KRT19 is directly regulated by KLK5 and to clarify the functional relevance of this relationship. In addition, the cholesterol- and mTORC1-related pathways identified in our functional and proteomic analyses should be considered as promising candidate mechanisms rather than definitively established downstream pathways of KLK5. Their precise mechanistic contribution to KLK5-mediated tumor biology remains to be determined. Additionally, although the association of KLK5 with OS is significant, the results would benefit from validation in an independent cohort, as the cut-off definition and formal analysis were performed within the same cohort. Moreover, the association between KLK5 and OS loses significance in the multivariable analysis that includes other clinical factors, indicating that KLK5 is not an independent prognostic factor. These limitations provide important directions for future research. Larger patient cohorts and additional patient-derived models, together with targeted loss-of-function and mechanistic studies, will be necessary to further define the relationship between KLK5 and KRT19 and to determine the contribution of the identified metabolic and signaling pathways to KLK5-driven tumor progression.

## 6. Conclusions

In summary, our clinical and experimental findings support the role of KLK5 as a tumor-promoting factor in HGSOC. High KLK5 expression was significantly associated with shorter overall survival in our patient cohort, while pronounced KLK5 overexpression reduced survival in a syngeneic mouse model. Together, these findings link KLK5 to an unfavorable clinical outcome and provide functional evidence that it actively contributes to ovarian cancer progression. Rather than acting as a linear inducer of a single effector, KLK5 appears to coordinate several partially independent tumor-promoting programs. Although our initial rationale centered on the association between KLK5 and KRT19, and KLK5 was confirmed as the KLK4–7 family member most consistently associated with KRT19 upregulation in vitro, in vivo, and in patient tumor tissue, KLK5 recapitulated the KRT19 phenotype only in part, i.e., concordant in spheroid formation but discordant in ECM adhesion and directed migration. The partial overlap, together with proteomic remodeling of the cholesterol/mevalonate pathway and the mTORC1 machinery, and a reduction in the favorable prognostic chemokine CXCL9 with decreased CD3+ infiltration, indicates that KRT19 induction is one component of a broader, context-dependent KLK5 program spanning cytoskeletal plasticity, metabolic reprogramming, and immune modulation. These associations remain to be causally dissected: the direct mechanistic links between KLK5 and KRT19 regulation, CXCL9 suppression, and mevalonate-pathway activation, as well as the dependence of these effects on KLK5’s catalytic activity, require dedicated mechanistic and interventional studies. This study provides a comprehensive analysis integrating diverse tumor-promoting processes associated with KLK5 in HGSOC and aims at elucidating pathways that might be therapeutically or diagnostically addressable. Collectively, our findings support the further evaluation of KLK5 as a prognostic biomarker and potential therapeutic target. If the identified mechanisms are confirmed, KLK5-directed strategies could be explored in KLK5-high HGSOC, potentially in combination with mevalonate-pathway inhibition or immune checkpoint blockade.

## Figures and Tables

**Table 1:** Multivariate analysis of the association between KLK family members and KRT19 expression in the TCGA ovarian cancer cohort (*n* = 426).

Multivariable Analysis ^(a)^			
Variable	β (Estimate)	*p*-Value	VIF ^(b)^
KLK4	−0.03	0.81	1.01
KLK5	0.060	0.0097	1.34
KLK6	0.047	0.15	1.46
KLK7	0.142	0.00024	1.71

^(a)^ R^2^ = 0.13; ^(b)^ Variance inflation factor (VIF) values < 2 indicate no relevant multicollinearity among predictors.

**Table 2:** Multivariable Cox proportional hazards regression models for overall survival and progression-free survival, including ascites, residual tumor burden, age, and KLK5 expression; hazard ratios, 95% confidence intervals, and *p* values are shown.

**Overall Survival**		
	**CI**	***p*** **-Value**
Ascitic fluid	1.387 (0.86–2.24)	0.182
Residual tumor	2.543 (1.65–3.92)	0.001
Age	1.324 (0.90–1.95)	0.157
KLK5	1.326 (0.80–2.21)	0.279
**Progression-Free Survival**		
	**CI**	***p*** **-Value**
Ascitic fluid	1.955 (1.31–3.38)	0.016
Residual tumor	1.729 (1.11–2.70)	0.016
Age	1.640 (1.07–2.51)	0.022
KLK5	1.177 (0.67–2.06)	0.569

## Data Availability

The data generated and analyzed during the current study are available from the corresponding author upon reasonable request. Patient-level clinical data are not publicly available due to ethical and data protection restrictions. The publicly available TCGA ovarian cancer dataset analyzed in this study can be accessed through [GDC Data Portal/cBioPortal; accessed 10 April 2026; available at https://www.cbioportal.org/study/summary?id=hgsoc_tcga_gdc].

## Supplementary Materials

The following supporting information can be downloaded at: https://www.mdpi.com/article/10.3390/cancers18203387/s1, Table S1: characteristics of the HGSOC cohort; Table S2: Original data; Supplementary Figure S1: Influence of KLK5 on Spheroid growth, Supplementary Figure S2: Proliferation of OV-MZ-6, Supplementary Figure S3: Trans-well Migration of OV-MZ-6 and Kuramochi KLK5 and empty vector cells through 5 μm pores, Supplementary Figure S4: Validation of KLK5 overexpression on the protein level.

## Abbreviations

The following abbreviations are used in this manuscript:

Col IV	Collagen IV
KRT	Cytokeratin
ELISA	Enzyme-linked immunosorbent assay
ECM	Extracellular matrix
FDR	False discovery rate
FCS	Fetal calf serum
FN	Fibronectin
GSEA	Gene set enrichment analysis
HGSOC	High-grade serous ovarian cancer
HRP	Horseradish peroxidase
KLK	Kallikrein-related peptidase
NES	Normalized enrichment score
PAR	Protease-activated receptor
SFM	Serum-free medium
SEM	Standard error of the mean
uPAR	Urokinase-type plasminogen activator receptor
VIF	Variance inflation factor
VN	Vitronectin
